# Immunomodulatory Effects of Vitamin D Receptors and Serum Vitamin D Levels in COVID-19 Patients After Immunization: A Narrative Review

**DOI:** 10.3390/ijms27209354

**Published:** 2026-10-21

**Authors:** Jaishriram Rathored, Sandesh Shende

**Affiliations:** Central Research Laboratory and Molecular Diagnostic, Allied Health Sciences, Datta Meghe Institute of Higher Education and Research, Sawangi (Meghe), Wardha 442001, Maharashtra, India; sandeshshende96@gmail.com

**Keywords:** immunization, immune mechanisms, inflammatory control, vaccine responsiveness, immunomodulation

## Abstract

Coronavirus disease 2019 (COVID-19) has caused global morbidity and mortality. Vaccination remains the most effective preventive strategy. However, variation in immune response after immunization has been observed among individuals. vitamin D plays a crucial role in immune regulation through activation of the VDR, which is expressed on various immune cells. The role of vitamin D status in post-vaccination immune responses among COVID-19 patients has gained increasing scientific interest. This narrative review evaluates the immunomodulatory roles of vitamin D receptors (VDRs) and serum vitamin D status in COVID-19 patients following vaccination, with particular emphasis on immune mechanisms, inflammatory regulation, and vaccine-induced immune responses. The relevant literature concerning vitamin D, VDR signaling, COVID-19 infection, vaccination, and immune responses was comprehensively reviewed. Emphasis was placed on studies discussing immune modulation, cytokine regulation, and antibody responses. Current evidence suggests that adequate serum vitamin D status may support both innate and adaptive immune responses following COVID-19 vaccination. Activation of the VDR regulates T-cell differentiation, modulates pro-inflammatory cytokine production, and supports humoral immune responses. Vitamin D deficiency has been associated with impaired vaccine responsiveness and persistent systemic inflammation. Vitamin D and VDR-mediated signaling pathways appear to play important roles in regulating immune responses following COVID-19 vaccination. Maintaining optimal vitamin D levels may help improve vaccine-induced immunity and reduce excessive inflammatory responses.

## 1. Introduction

The COVID-19 pandemic has fundamentally changed the global perception of public health, infectious diseases, and preventive medicine. Since the first cases, the disease caused by the severe acute respiratory syndrome coronavirus 2 (SARS-CoV-2) has impacted millions of people all over the world and regardless of their age and location [1]. Successive waves of infection have put the health systems under severe pressure, and the quotidian life has been shaken. Scientists and healthcare authorities, in turn, have strived to create safe and effective medicines at an unprecedented rate. Implementation of COVID-19 vaccination programs has played a significant role in reducing severity of disease, hospitalization, and deaths. However, it was soon realized that after vaccination, not all people have the same immune response. Some individuals develop robust and long-lasting immunity, whereas others generate relatively weaker immune responses. This heterogeneity is also affected by factors such as age, existing medical conditions, heredity, nutritional status and general state of immune health. Of these determinants, nutrition, especially micronutrients, has received increased attention. Vitamin D is one of the micronutrients under growing investigation as an immune regulatory nutrient in infection with SARS-CoV-2 and after vaccination [2].

Vitamin D is a fat-soluble secosteroid hormone that is well recognized for maintaining calcium homeostasis and skeletal health. Over the past two decades, accumulating evidence has demonstrated that vitamin D also plays an important role in immune regulation. Vitamin D deficiency is recognized as a major global public health concern. Vitamin D deficiency results from multiple factors, including urban lifestyles, limited sunlight exposure, air pollution, increased skin pigmentation, reduced outdoor activity, and inadequate dietary intake [3]. Several studies have demonstrated that a low serum vitamin D level is linked to increased vulnerability of respiratory tract infection, inefficient immune reaction, and inflammation. Observational studies conducted during the COVID-19 pandemic have consistently reported that people who were vitamin D deficient were more prone to severe disease, hospitalization or poor outcomes. These observations have triggered the desire to explain vitamin D regulation of immune activities. Vitamin D exerts its biological effects primarily through activation of the VDR located on many immune cells such as macrophages, dendritic cells, T lymphocytes, and B lymphocytes. The ability to bind vitamin D to VDR regulates immune function, reduce excessive inflammation and strengthen protective immunity [4].

However, when it comes to COVID-19 vaccination, the role of vitamin D takes an even more crucial role. Vaccines work by causing the immune system to produce antibodies and memory cells which provide defense against future infection [5]. The process requires an optimal level of immune reaction where the host is able to generate adequate protection without having to induce a harmful inflammatory effect. Vitamin D, via its interface with VDR, could help to maintain this balance, both via innate and adaptive immunity. Answering the question of how serum vitamin D levels and VDR activity affect post-vaccination immune responses might give helpful results on improving the efficiency of vaccines and patient outcomes [6]. It is especially relevant to countries like India, where vitamin D deficiency is widespread and the rate of COVID-19 vaccination is widespread. An in-depth knowledge of this interaction can help to identify people who are at risk of poor vaccine response and directing the supportive interventions, such as nutritional optimization.

This review, therefore, aims at investigating how VDRs and serum vitamin D levels, in COVID-19 patients when immunized, immunomodulate immune responses, and whether they influence the post-vaccination immune responses.

## 2. Literature Search Strategy

This narrative review was conducted to summarize current evidence regarding the role of VDR signaling and serum vitamin D status in modulating immune responses following COVID-19 vaccination. Although this review does not follow a systematic review methodology or PRISMA guidelines, a structured literature search was performed to improve transparency and comprehensiveness.

Electronic databases including PubMed, Scopus, Web of Science, and Google Scholar were searched for studies published between January 2004 and June 2026. Search terms included combinations of: “Vitamin D”, “VDR”, “VDR”, “COVID-19”, “SARS-CoV-2”, “COVID-19 vaccination”, “Vaccine immunogenicity”, “Immune response”, “Humoral immunity”, “Cell-mediated immunity”, “Antibody response”, “Cytokines”, “Inflammation”

Reference lists of relevant review articles and original studies were also manually screened to identify additional eligible publications.

The review included peer-reviewed original research articles, randomized controlled trials, observational studies, cohort studies, case–control studies, systematic reviews, meta-analyses, and mechanistic experimental studies published in English. Editorials, conference abstracts, duplicate publications, and studies unrelated to vitamin D-mediated immune regulation or COVID-19 vaccination were excluded.

Evidence was narratively synthesized according to major themes, including vitamin D metabolism, innate immunity, adaptive immunity, cytokine regulation, COVID-19 disease severity, vaccine immunogenicity, VDR polymorphisms, supplementation studies, and future research directions.

## 3. Vitamin D Metabolism and VDRs Biology

### 3.1. Vitamin D Metabolism

Vitamin D is an essential micronutrient that plays critical roles in skeletal integrity and immune system regulation. The major source of vitamin D in humans is sunlight; ultraviolet B radiation causes photochemical transformation of 7-dehydrocholesterol in human skin (epidermis) into cholecalciferol (vitamin D 3) [7]. In addition to cutaneous synthesis, dietary sources like fatty fish, egg yolk, liver, fortified milk, fortified cereals and nutritional supplements also contribute to circulating vitamin D concentrations. Among the Indian population, though, the consumption of vitamin D is generally not the best due to the predominantly vegetarian diets which are not highly enriched with vitamin D [8]. Cutaneous synthesis remains the principal source of vitamin D in humans. Despite abundant sunlight, vitamin D deficiency remains highly prevalent in India. Factors include the current lifestyle practices including prolonged indoor living, lack of physical exercise, usage of covering clothes, use of sunscreen regularly and increased air pollution in the environment, which decrease the effective cutaneous exposure to UVB [3]. Moreover, increased pigmentation of the skin also suppresses the synthesis of vitamin D, whereby a darker dermis needs a more extended period of sun exposure to synthesize the same amounts of vitamin D [9]. The outcome is a high percentage of the population, such as pediatric, adult and geriatric cohorts, having a sub-normal vitamin D concentration. When vitamin D enters the organism, it requires two consecutive metabolic processes to become biologically active, either through the cutaneous or gastrointestinal route [10]. The hepatic first stage is the hydroxylation of vitamin D to 25-hydroxyvitaminD (25 (OH)D), a circulating form of this vitamin and the standard form of metabolite regularly measured to determine vitamin D status. The latter process occurs in the renal cortex, whereby 25(OH)D is hydroxylated to produce the hormonally active 1,25-dihydroxyvitamin-D (1, 25(OH) 2D) [11]. The physiological activities of vitamin D are mediated by this form of the vitamin. It is notable that a number of immune cells such as the macrophages and dendritic cells have the enzymatic machinery needed to convert 25(OH)D to the active form locally, thus allowing vitamin D to regulate immune activities independently in lymphoid tissues either during infectious events or after vaccination [12]. Together, vitamin D and VDR are crucial for preserving immune system equilibrium. Serum phosphorus and parathyroid hormone, which are essential for calcium homeostasis and cellular growth, control the synthesis and metabolism of vitamin D in the skin and kidney, as illustrated in Figure 1. In the process of such local activation, vitamin D affects the autocrine and paracrine effects, acting on the same cells that produce it or on neighboring populations of cells. In addition to immune regulation, vitamin D is important for the maintenance of musculoskeletal health, the regulation of cellular growth, cardiovascular health, and the maintenance of the integrity of epithelial barriers [13]. Collectively, these findings indicate that vitamin D functions as a pleiotropic hormone rather than merely a dietary vitamin.

### 3.2. VDR Biology

The biological actions of vitamin D are primarily mediated through the VDR, a nuclear receptor of the large family of hormone-responsive transcription factors that coordinate the expression of genes. Structurally, VDR contains distinct ligand-binding and DNA-binding domains that regulate gene transcription. When 1, 25(OH) 2D enters a target cell, the ligand binds nuclear VDR, which forms a heterodimer with the retinoid X receptor [15]. This receptor complex is in turn bound to certain segments of DNA called vitamin D response elements (VDREs). The binding to these sequences regulates transcriptional activation or repression of a broad range of immune regulation, inflammatory regulation, cell differentiation, and survival genes [16]. A characteristic feature of VDR is its ubiquitous tissue expression; in addition to traditional destinations like bone, intestine, and kidney, VDR is also expressed in immune cells. In the macrophages, which are the main innate defense cells, VDR stimulation enhances microbial killing receptors and suppresses pro-inflammatory signaling [17]. Tolerant VDR expression on dendritic cells that mediate antigen presentation and T-cell priming also regulates vitamin D signaling that suppresses excessive maturation of dendritic cells and maintains immune tolerance and immunological homeostasis. Adaptive immunity is mediated by T lymphocytes, which upregulate VDR upon activation and the receptor regulates T-cell differentiation so that the balance between pro-inflammatory and regulatory immune responses is maintained [18]. VDR is also expressed in B cells or (B) lymphocytes which generate humoral antibodies and vitamin D promotes cell growth, differentiation, and antibody production. The extensive distribution of VDR in the immune system of the body and the main functions of vitamin D in direct and active control of immune regulation is significant [19]. VDR-mediated signaling gains the primary prominence in the framework of the viral infection, including COVID-19, and the post-immunization period. The ideal VDR activity will provide an adequate level of immune response to provide protection but will be strictly controlled to prevent harmful inflammation. Differences in the expression or the functionality of VDR, in combination with low vitamin D in the blood, can therefore impact human susceptibility and reaction to infectious disease and to immunization [20].

## 4. Immunomodulatory Mechanisms of Vitamin D

### 4.1. Effects on Innate Immunity

Vitamin D has a central effect on innate and adaptive immunity in a variety of mechanistic pathways. The first defense mechanism in opposition to pathogens is the innate immune system, which happens as soon as it is exposed to disease or vaccination [21]. Vitamin D enhances innate immune responses, increasing the production of antimicrobial peptides cathelicidin and defensins. These peptides also have direct antiviral and antibacterial properties, which suppress the initial viral replication. Also, they strengthen epithelial barriers in the respiratory system, creating an innate physical barrier to the invasion of the virus [22]. Vitamin D is also vital in regulating the macrophages and natural killer cells. It maintains a balance by strengthening their ability to sense and remove infected cells and at the same time limits excessive inflammatory responses, which can result in the development of severe pulmonary damage and its subsequent complications: in COVID-19, unregulated inflammation can lead to critical pulmonary injury [12].

### 4.2. Effects on Adaptive Immunity

Adaptive immunity, characterized by antigen-specific immune responses, is also immensely affected by vitamin D. Its immunomodulatory effects primarily involve the major effectors of adaptive immunity, T cells and B cells. Vitamin D inhibits the hyperactivity of Th1 and Th17 subsets, which release pro-inflammatory cytokines; hyperactivity of these subsets has been associated with the widespread tissue injury of COVID-19 [23]. Simultaneously, vitamin D enhances the maturation of regulatory T cells and, hence, the development of immune tolerance and the inhibition of immune over-response. Vitamin D also facilitates proper differentiation and regulation of antibody production in B cells a necessary requirement in a strong post-vaccination response [24].

### 4.3. Regulation of Cytokine Responses

The other important immunological role of vitamin D is that it regulates the production of cytokines. Cytokines are signaling molecules that coordinate immune reactions. The unchecked release of pro-inflammatory cytokines that is usually described as a hyper-inflammatory cytokine response (“cytokine storm”) is closely linked to negative outcomes in severe COVID-19 infection [25]. VDR plays a role in regulating the generation of cytokines like interleukin-6 and tumor necrosis factor-α, and at the same time, enhances anti-inflammatory cytokines. This cytokine balance is particularly crucial after immunization, when vaccination requires activation of an effective immune response, but at the same time, it cannot provoke a strong inflammatory response [26].

### 4.4. Vitamin D and Host Defense Against Viral Infections

The topic of vitamin D and different viral respiratory infections due to influenza and other coronaviruses have also been studied. It is linked to low vitamin D which is associated with increased susceptibility and severity to these infections [27]. With regard to COVID-19, vitamin D can play a role in viral entry, replication and the immunopathogenic pulmonary injury that follows. Vitamin D may help alleviate the severity of the disease and speed up the recovery process by maintaining the integrity of epithelial cells, balancing the immune cells, and reducing the inflammatory response.

Adequate vitamin D levels and functional VDR signaling can be used to ensure the vigorous but balanced immune response in individuals that have been vaccinated, thus increasing the effectiveness of the vaccine and reducing negative immunologic responses [28]. VDR activation suppresses several pro-inflammatory signaling pathways, particularly nuclear factor-κB (NF-κB), while promoting anti-inflammatory cytokine production, including interleukin-10. These molecular effects contribute to maintaining immune homeostasis and preventing excessive inflammatory responses following SARS-CoV-2 infection and vaccination. Experimental studies further demonstrate that VDR signaling regulates antigen presentation, dendritic cell maturation, macrophage polarization, and T-cell differentiation, thereby coordinating both innate and adaptive immune responses [29].

## 5. Serum Vitamin D Status in COVID-19 Patients and Immune Dysfunction

In the COVID-19 pandemic, an array of clinical and epidemiological studies in various geographic locations reported that a significant percentage of infected persons had sub-optimal levels of 25-hydroxyvitamin-D in their serum [30]. The 25-hydroxyvitamin D levels measured as a valid surrogate of vitamin D status were deficient throughout the range of disease severity from mild to moderate and to severe COVID-19. Such inadequacy was found especially among the elderly and individuals with chronic comorbidities like diabetes mellitus, arterial hypertension, obesity, and renal impairment and also in individuals that have limited photic exposure [31].

The clinical situation in India was even more alarming since the vitamin D deficiency was already internalized in the rest of the population before the pandemic [3]. The lockdown at the national level, the overall use of remote working, the shutdown of outdoor recreational facilities, and the fear of congregational exposure of the population all worked together to reduce the exposure to sunlight among significant parts of the population. Therefore, a significant number of people were starting the pandemic with dwindling amounts of vitamin D [32].

A study carried out in hospitals located in India reported that most of the people admitted with COVID-19 had inadequate or lacked vitamin D. This epidemiologic indicator posed strong questions about whether a decreased vitamin D position may either put people at risk of infection or worsen the course of the disease [33,34].

Even though vitamin D deficiency alone cannot explain the COVID-19 pathogenesis, its high prevalence among its victims indicates that it could potentially mediate the host response to the virus. Vitamin D promotes maintaining the balance of the immune system, and its deficiency possibly reduces the immune system to an inadequate defense against new viral invaders [35]. The systematic finding of low vitamin D in heterogeneous groups and both clinical and non-clinical manifestations of the disease has highlighted the importance of using the vitamin D status to question the COVID-19 epidemiology, especially in countries like India, where deficiency is widespread across all age groups [36].

An emerging literature streamlines an effective correlation between vitamin D serum levels and the COVID-19 disease clinical severity [37]. A study cited that patients with lower levels of vitamin D were unfairly associated with the development of severe manifestation, the need to be placed on supplemental oxygen or mechanical ventilatory, the need to be admitted in intensive care, or the need to take lengthy courses in hospitals. The mortality rate was higher in some of the cohorts which were deficient in vitamin D as compared to their counterparts who were not deficient in vitamin D.

A salient mechanistic explanation of such a correlation is that vitamin D has a regulatory effect on inflammation. Strong COVID-19 infection is often typified by an overreaction of the inflammatory environment, which is marked by a high concentration of C reactive protein, interleukin-6, ferritin, and D-dimer in the blood [38]. It has been indicated that vitamin D deficiency is associated with increased levels of such biomarkers, which are a sign of unregulated and uncontrolled inflammation. When the vitamin D status is insufficient in individuals, the immune system tends to become pro-inflammatory, and a stronger Th1 and Th17 lymphocyte subsets activation has been observed [39]. These cells release a large number of inflammatory cytokines capable of causing harm to pulmonary parenchyma and other vital organs. At the same time, the negative regulation mechanisms which normally check overexerted inflammation are muted in the vitamin D-deficient conditions [40].

There is a possibility that the low levels of vitamin D do not operate independently and in combination with other risk factors like old age, obesity and chronic disease, the low levels of vitamin D are synergist with other risk factors and hence compromised immunity functions. Taken together, these interrelations can increase the risk of the development of severe disease, extend the process of clinical recovery, and make convalescence more complicated [41].

The vitamin D deficiency does not only increase inflammation, but it also disrupts both innate and adaptive lymphocytic responses, leading to an overall disruption of immune homeostasis. In the inborn arm, the vitamin D deficiency reduces the antimicrobial peptides production, e.g., cathelicidin and defensins, that restrain the initial viral replication [23]. The attenuation allows the virus to proliferate freely, leading to increased viral loads and severity of clinical symptoms. Other key players of innate immunity such as the macrophages and natural killer cells show impaired functionality in people lacking vitamin D, which further increases the burnt on the adaptive immune system and compromises the initial removal of the virus [42].

Vitamin D deficiency in the adaptive immune compartment suppresses the action of regulatory T cells which are most vital in immune tolerance and balance. At the same time, it changes B-cell development and activity, which may compromise the quality and sustainability of humoral responses [43]. This immunological disequilibrium has a particularly significant effect on the period of convalescence and in the post-immunization period, when the presence of strong antibody response and the formation of immune memory is essential [44]. Sufficient vitamin D can be used to overcome inflammation by quelling the pro-inflammatory cytokine production and promoting tissue repair networks. The empirical data show that patients who had a supply of vitamin D healed faster and with a lower number of sequelae after COVID-19 [45].

One of the new directions of vitamin D in long COVID-19 is a syndrome with manifestations such as persistent fatigue, dyspnea, and neurocognitive impairment. The underlying factors that have been hypothesized to contribute to these sequelae are chronic and low-grade inflammation and long-term immune activation and vitamin D may have a restorative effect in restoring immune homeostasis over time [46]. Clinically, vitamin D deficiency is a risk factor that is variable. Frequent monitoring of vitamin D levels in patients with COVID-19 and prompt repair of deficiency may help achieve more positive immune responses. Regarding vaccination, adequate levels of vitamin D before or after vaccination could be beneficial in terms of quality of the vaccination-induced immune response and long-term protection. Although conclusive controlled trials are necessary to support these findings, the body of evidence highlights the significance of ensuring adequacy in the levels of vitamin D as part of the overall immune strength in the present and after COVID-19 infection.

## 6. Evidence Quality and Methodological Considerations

Current evidence investigating vitamin D status and post-vaccination immune responses consists predominantly of observational studies, retrospective cohort studies, and a limited number of randomized controlled trials. Consequently, interpretation of available findings requires careful consideration of methodological limitations [47].

Several important confounding variables may influence reported associations between vitamin D status and vaccine immunogenicity. These include participant age, obesity, diabetes mellitus, baseline nutritional status, prior SARS-CoV-2 infection, vaccine platform (mRNA, viral vector, or inactivated vaccines), timing of vitamin D measurement, seasonal variation, assay methodology, ethnicity, sunlight exposure, and vitamin D supplementation history [48].

While observational studies consistently report associations between vitamin D deficiency and impaired immune responses, these studies cannot establish causality. Randomized controlled trials evaluating vitamin D supplementation before or after COVID-19 vaccination have produced inconsistent findings, suggesting that supplementation may primarily benefit individuals with documented vitamin D deficiency rather than vitamin D-sufficient populations [49].

Overall, current evidence should therefore be interpreted as demonstrating an association rather than definitive causal evidence. Larger multicenter randomized controlled trials with standardized supplementation protocols and long-term immunological follow-up are required.

## 7. Role of Vitamin D in COVID-19 Vaccination and Immune Response

COVID-19 vaccination is considered to be one of the most important interventions of the current war on public health because it aims to limit the spread and clinical severity of SARS-CoV-2. The different systems’ mRNA, viral vector, and inactivated virus formulations have been designed, but all aim to promote the adaptive immune response in recognizing the viral spike protein, and to develop a quick and specific response to the virus each time exposed in future [50]. After vaccination, the antigen-presenting cells internalize a vaccine-derived antigen into the cell, like dendritic cells and macrophages; the cells process and present peptides through MHC class I or II molecules to T lymphocytes. This leads to the activation of helper and cytotoxic T cells that cause a multi-pronged defense: helper T cells promote B-cell class switching and antibody production, whereas cytotoxic T cells destroy infected cells. At the same time, memory B and T cell compartments are produced and this provides progressive protection against severe disease [51]. However, empirical findings indicate that post-vaccination immune responses are heterogeneous; it is always reported that there is inter-individual variation in humoral and cellular measurements. The demographic and clinical ones, such as age, sex, nutritional conditions, obesity, diabetes, and other comorbidities, have quantifiable impact on vaccine-induced immunity [52]. Immunosenescence causes older adults to have attenuated antibody titers and reduced reactivity in T cells. Equally, patients, whose immunocompromised states prevent them, do not develop effective responses. Of the factors related to nutrition, vitamin D deficiency has proven to be a viable cause of such disparities, considering the proven immunoregulatory effect of vitamin D. The presence of low circulating levels of 25-hydroxyvitamin D can potentially undermine the quality and size of the vaccine response, which is why it is crucial to explain these differences to streamline immunization efforts and maximize the protection of vulnerable groups [53].

Vitamin D has also gained major concern due to its putative effect in enhancing vaccine-induced immunity. One of the most important mechanistic routes is a signal modulating antibody production: B-cell stimulation and class switch recombination is optimized by a set of signals transmitted by helper T cells and cytokines, which can be refined by vitamin D by maintaining immune homeostasis and suppressing inappropriate inflammation [54]. An observational study has observed that serum 25-hydroxyvitamin D levels are positively correlated with post-vaccination anti-SARS-CoV-2 antibody titers, which is consistent with other antigenic challenges. But these associations do not uniformly repeat, probably because of the complex interaction of host factors, vaccine platform and immunological milieu [55]. In addition to humoral immunity, T cell-mediated immunities play a specific role in creating long-term immunity especially when the antibodies are depleted with time. Vitamin D promotes a regulatory environment through T-regulatory cell differentiating and saluting Th1/Th17 cell activities, thus maintaining a balanced antiviral response. The balance post-vaccination lies here: it is necessary to have a strong but restrained immune response that will form immunological memory without causing cytokine storms that are detrimental. Vitamin D can potentially help stimulate a stronger and better lasting immune response by simultaneously boosting the cellular and antibody response. Even though large, randomized trials are necessary to ascertain causality, the existing body of literature has a collective indication that vitamin D deficiency may be used to maximize immunogenicity after COVID-19 immunization [56].

Relevance of vitamin D is also enhanced in light of receptor-mediated effects and inflammatory pathways after vaccination. Antigen-stimulated immune cells increase the expression of VDR after immunization; ligand-bound VDR complexes regulate the expression of major genes that regulate the production of cytokines, cell survival, and antigen presentation [12]. This controlled gene that relies on VDR can make the immune activation brought about by vaccination effective but proportional. VDR signaling also overlaps with fundamental inflammatory pathways, especially the nuclear factor kappa B (NF-kB) pathway, which is a mediator of pro-inflammatory cytokines [29]. Vitamin D suppresses overaggressive cytokine signaling by inhibiting NF-kB activation, inhibiting possible post-vaccination inflammatory aftermaths in the form of fever or malaise. Such anti-inflammatory regulation proves especially beneficial in those who have been infected with SARS-CoV-2 previously, and their immunity can be fitted and preconditioned to hyper-react. Sufficient vitamin D status is thus likely to increase vaccine tolerability, lower inflammatory side effects and facilitate a more successful recovery of immune homeostasis. Deficit, on the other hand, can predispose to inadequate vaccine reactions, prolonged immune dysregulation, and reactogenicity that is particularly high in elderly people and those with a chronic condition [5]. Figure 2 shows a schematic diagrammatic demonstration of VDR pathway-mediated immune regulation and cytokine modulation. Overall, the preservation of adequate vitamin D levels promotes balanced immune responses after immunization, which is likely to enhance efficacy, safety, and overall outcomes in massive immunization influencing campaigns [19].

## 8. VDR Polymorphisms and Personalized Immune Responses

Genetic polymorphisms of the VDR, including FokI, BsmI, ApaI, and TaqI, influence receptor activity, ligand binding, and downstream gene transcription. Emerging evidence suggests that these polymorphisms contribute to inter-individual variability in immune responses, susceptibility to infectious diseases, and vaccine responsiveness [57].

Several observational studies have reported associations between specific VDR genotypes and COVID-19 severity, although evidence regarding vaccine-induced immune responses remains limited. Individuals carrying low-function VDR variants may require higher circulating vitamin D concentrations to achieve comparable immunomodulatory effects [58].

Future precision medicine approaches integrating serum vitamin D concentrations with VDR genetic profiling may improve individualized vaccination strategies and targeted nutritional interventions.

## 9. Vitamin D and VDRs COVID-19 Vaccination Clinical Evidence

Since the introduction of COVID-19 vaccines, observational study has examined the hypothesis that circulating levels of 25-hydroxyvitamin D regulate the post-vaccination repertoire. These trials, including a variety of cohorts, evaluated endpoints, including particular antibody titers, inflammatory surrogate markers, and breakthrough SARS-CoV-2 infection. In the majority of series, the neutralizing antibodies of people with adequate vitamin D status were significantly higher after the inoculation compared with deficient people [59]. Other reports also found that the vitamin D-deficient participants produced lower magnitude of the antibody with delayed kinetics. Taken together, these results indicate that the architecture of humoral immunity triggered by COVID-19 vaccine may be affected by the status of vitamin D [60]. Some of the studies did not find any significant association between the vitamin D status at baseline and the results of the vaccine-induced immune parameters. This heterogeneity probably arises due to methodological differences, such as the timing of vitamin D measurements and vaccine administration, the type of vaccine used, demographic and health characteristics of the study participants, and previous experience of SARS-COVID-2 [61]. As an example, antibody dynamics can change based on the pre-immunization, post-immunization and weeks post-immunization measurement of vitamin D. In addition, investigations that integrated members with diverse nutritional conditions also make it difficult to isolate the univariate impact of vitamin D. In spite of these limitations, the preponderance of the observational data suggests that adequate vitamin D status may support stronger vaccine-induced immune responses after vaccination, especially in areas where the deficiency is endemic. These notes demonstrate why nutritional assessment should be integrated as a part of the general assessment of vaccine responsiveness, particularly in the impoverished subgroups of the elderly and those with chronic illnesses.

A more definitive evaluation of the putative effect of vitamin D in vaccine-induced immunity can be conducted by interventional research and controlled supplementation trials, which are, nevertheless, relatively scarce. Vitamin D supplementation before or soon after COVID-19 vaccination was linked to enhanced antibody titers and reduced concentrations of inflammatory biomarkers in a subset of randomized trials. The beneficial effects were greatest among subjects who were vitamin D depleted at baseline; in this group, the restoration of the depleted stores was found to reestablish the immune milieu, with more controlled antibody-mediated response and alleviating the pro-inflammatory pathways [62]. On the contrary, the supplementation did not produce any significant improvement on immunologic endpoints in individuals who already had sufficient levels of vitamin D. This trend suggests that vitamin D treatment can be best utilized as a correctional measure but not an enhancing factor in individuals with normative samples. Safety-wise, the use of vitamin D supplement is otherwise well tolerated when taken within the prescribed therapeutic range [63]. Intravenous short-term regimens that have been established at the same time as vaccination have any major adverse events in clinical trials. However, hypercalcemia may occur as a result of hypervitaminosis D, and it is characterized by gastroenterological disorders, muscle weakness, and nephrotoxic outcomes [64]. Based on this, the supplementation should not be administered indiscriminately, but through accurate serum measurements and clinical guidance at high dosages in all patients. Such data support the idea that a special approach in which the supplementation will be considered based on the registered deficiency and the personal evaluation of risks.

Another essential aspect of the existing clinical evidence is the area of genetic heterogeneity of VDR and its immunologic implications. VDR polymorphisms regulate the receptor activity, and, thus, the cellular immunomodulatory response towards the circulating vitamin D [65]. There are specific VDR allelic variants associated with the immunomodulatory response and increased vulnerability to infectious diseases. In the COVID-19 scenario, this genetic variability can be part of the explanation of inter-individual differences in disease progression and vaccine reactivity in otherwise similar vitamin D levels [66]. Some genotypes might require more circulating vitamin D levels to result in the same downstream immunological responses, indicative of low receptor affinity or signal transduction activity. This genetic subtlety provides another complication to the vitamin D studies and emphasizes the role of individual immunologic support measures. Indian national studies, although small in scope, bring some specific valuable population-based knowledge. Coin gate reports on the Indian cohorts have continuously reported a high prevalence of vitamin D deficiency among COVID-19 patients and healthy adult vaccinated population. Positive correlation between sufficient levels of vitamin D and improved post-vaccination immunological indicators. These observations are especially relevant when viewed in conjunction with the heterogeneity of India, its lifestyle, its diet, and the extent to which it is deficient [67]. However, the existing body of evidence has a number of drawbacks, such as a higher proportion of observational studies, small sample sizes of interventional trials, imprecision of supplementation regimens, and lack of long-term follow-up. A solution to these gaps would require large and carefully designed trials with heterogeneous populations and long-term evaluation of long-term immune outcomes. To the extent that these data have yet to be produced, the available evidence suggests that appropriate levels of vitamin D should be maintained as a part of the comprehensive immune condition, particularly when widespread vaccination programs are executed.

## 10. Clinical Implications

The available evidence suggests that maintaining adequate vitamin D status may contribute to optimal immune regulation during COVID-19 vaccination. However, current evidence does not support routine high-dose vitamin D supplementation solely to enhance vaccine efficacy in the general population [68].

Vitamin D assessment may be particularly beneficial in elderly individuals, immunocompromised patients, individuals with obesity, chronic kidney disease, diabetes mellitus, or documented vitamin D deficiency. Correction of deficiency according to established clinical guidelines may improve overall immune health while avoiding unnecessary supplementation among vitamin D-sufficient individuals [69].

Future clinical recommendations should be based upon adequately powered randomized controlled trials evaluating both humoral and cellular immune responses following vaccination.

## 11. Discussion

This narrative review summarizes current evidence related to the role of vitamin D and its receptors in regulating immune responses in patients with COVID-19 after vaccination. The reviewed literature indicates that vitamin D is necessary not only to skeletal and metabolic balance, but also to immune balance [70]. Vitamin D regulates immune function through VDR-mediated signaling pathways that influence cytokine, immune cell activation, and inflammatory cascades. Table 1 describes the association between vitamin D status and COVID-19 disease severity. These effects are particularly relevant in the context of COVID-19 vaccination because vaccines are based upon a coordinated immune response to generate antibody and long-term memory [64]. Adequate levels of vitamin D seem to boost B-cell production of antibodies and promote balanced T-cell reactions, which strengthen long-term immunity. At the same time, vitamin D suppresses hyper-inflammation that may otherwise delay immune recovery in the case of a person with previous SARS-CoV-2 infection [23]. Table 2 summarizes vitamin D, VDR, and COVID-19 vaccine-induced immune responses. These observations are solidly mechanistically based on ubiquitous VDR expression on macrophages, dendritic cells, T cells, and B cells. VDR stimulation alters immunological tolerance, antiviral defense- and inflammatory restraint, which confirm vitamin D as an immuno-supportive endocrine nutrient, but not a dietary supplement [71,72]. However, the available evidence remains heterogeneous; certain studies demonstrate strong positive effects whereas others demonstrate the non-significant associations or none whatsoever. This can be as a result of fluctuations in the baseline vitamin D levels, genetic polymorphism of the VDR functionalities, different vaccine platforms, age stratification and the time of vitamin D testing. Although these contradictions do exist, the overall trend suggests that vitamin D adequacy enhances more favorable post-vaccination immune responses, especially in those population groups suffering from severe deficiency.

## 12. Public Health Implications

The deficiency of vitamin D is common, is preventable and can be amended in a public health perspective. In India and other developing countries with low- and middle-income levels, improving the status of vitamin D by education, protection against the sun, fortification of food, and special supplementation may strengthen the collective immune system. The inclusion of simple nutritional screening in vaccination and preventive health programs can increase the overall outcomes in pandemics and future health crises.

## 13. Limitations

Being a form of narrative synthesis, it lacks a systematic methodology, which rules out exhaustive rejection of selection bias. Causal inference is also limited by the fact that most studies use observational studies. Other issues that also affect comparability across investigations are variability in the study design, sample size, and outcome measures.

## 14. Future Directions

Future study must put more emphasis on large and well-designed randomized controlled studies that will definitely determine the effect of vitamin D supplementation on vaccine-induced immunity. To understand immune memory and the efficacy of protection with time, longitudinal research is needed. Studies of VDR polymorphisms and population-specific advice will help to convert these results into the effective and targeted strategies.

## 15. Research Gaps and Future Perspectives

Despite increasing interest in vitamin D-mediated immunoregulation, several important knowledge gaps remain. First, randomized controlled trials evaluating vitamin D supplementation before COVID-19 vaccination remain limited in both sample size and methodological consistency. Second, standardized definitions of vitamin D deficiency and uniform measurement protocols are lacking across studies. Third, few investigations have simultaneously evaluated humoral immunity, cellular immunity, cytokine responses, and long-term immunological memory.

Furthermore, limited evidence exists regarding the interaction between VDR polymorphisms and vaccine-induced immune responses. Future multicenter longitudinal studies integrating molecular biomarkers, genetic profiling, immune phenotyping, and standardized supplementation protocols are needed to clarify the causal role of vitamin D in vaccine immunogenicity.

## 16. Conclusions

Vitamin D and VDR signaling play important immunomodulatory roles in regulating innate immunity, adaptive immunity, cytokine production, and inflammatory homeostasis during SARS-CoV-2 infection and following COVID-19 vaccination. Current evidence indicates that adequate vitamin D status is associated with more balanced immune responses and may contribute to improved vaccine immunogenicity in vitamin D-deficient individuals. However, the overall evidence remains heterogeneous, with most available studies being observational and therefore unable to establish causality. Randomized controlled trials investigating vitamin D supplementation have reported mixed findings, highlighting the need for standardized clinical studies. Future research integrating vitamin D status, VDR polymorphisms, molecular biomarkers, and long-term immunological outcomes will improve understanding of individualized immune responses and facilitate evidence-based recommendations for optimizing vaccine-induced immunity.

## Figures and Tables

**Figure 1:**
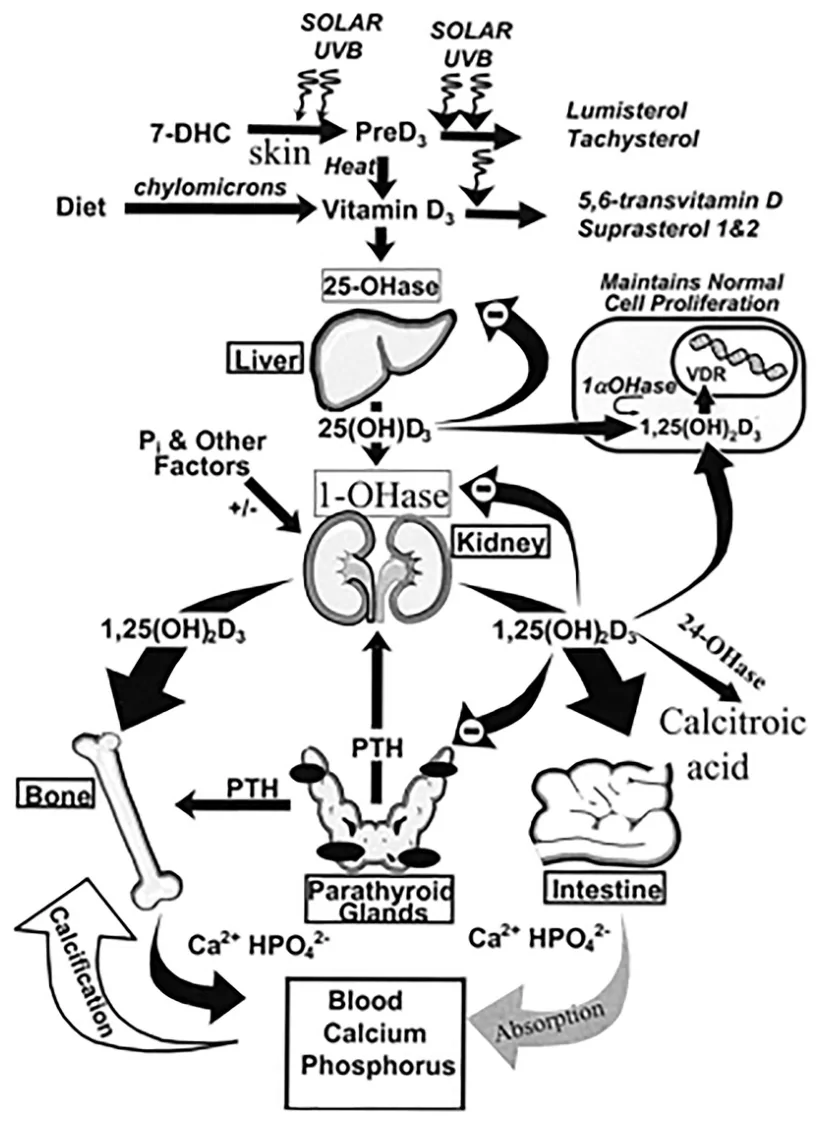
Vitamin D production and metabolism in the skin and kidney, important for calcium homeostasis and cellular growth (adapted from Holick et al.) [14].

**Figure 2:**
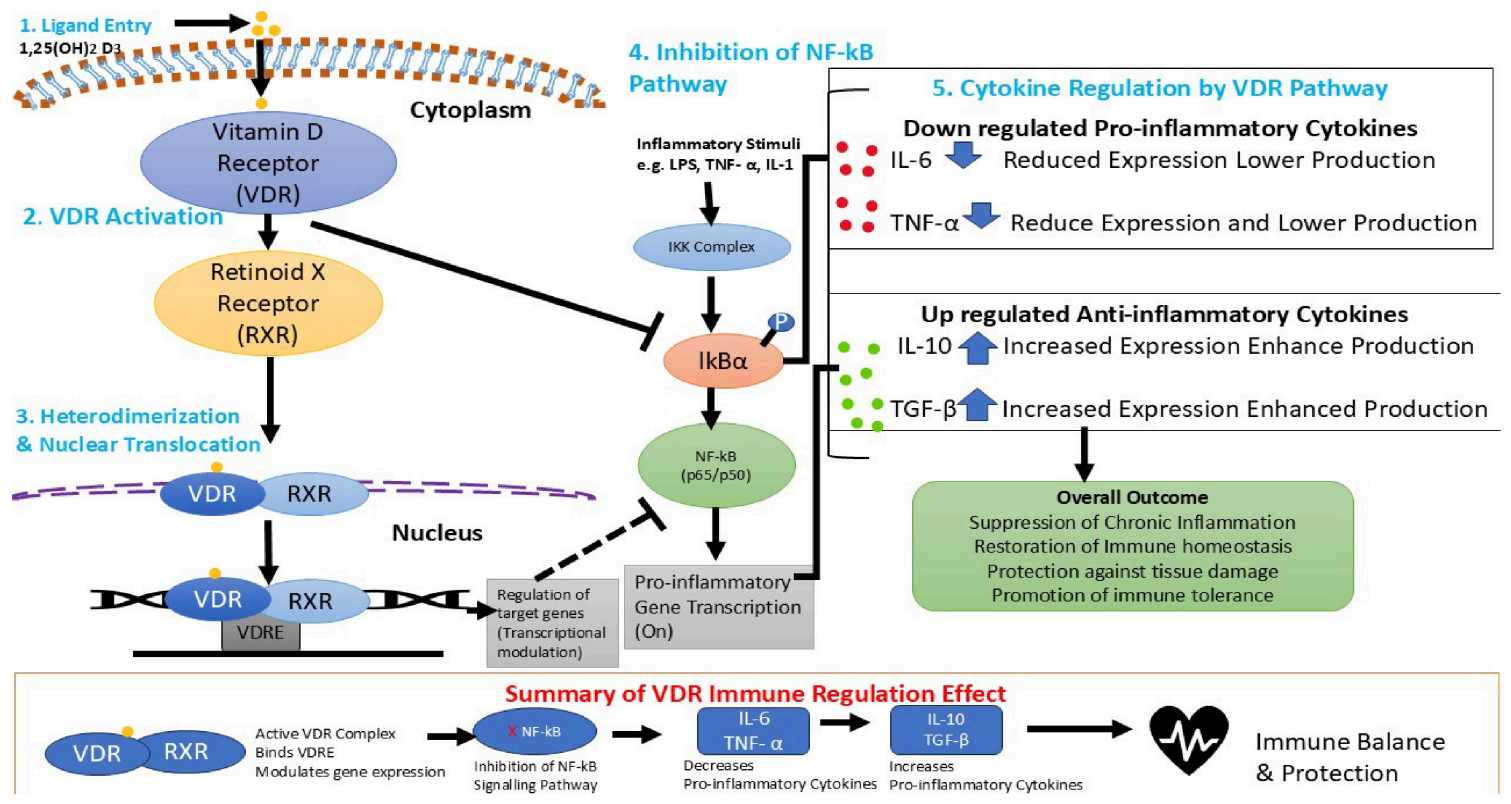
Schematic representation of VDR pathway-mediated immune regulation and cytokine modulation (made by author).

**Table 1:** Clinical studies evaluating the association between vitamin D status and COVID-19 disease severity.

Researcher(s) Name and Year	Finding of the Study	Merits	Demerits
Liu et al., 2021 [73]	Found that low vitamin D status is associated with adverse COVID-19 outcomes.	Comprehensive meta-analysis highlighting the importance of vitamin D in COVID-19 severity.	Does not directly address post-vaccination immune responses.
Bae et al., 2022 [74]	Reported an association between vitamin D status and COVID-19 severity.	Systematic review providing evidence on vitamin D’s role in COVID-19 outcomes.	Focuses on infection severity rather than post-vaccination immunity.
Israel et al., 2022 [75]	Identified vitamin D deficiency as associated with higher risks for SARS-CoV-2 infection and COVID-19 severity.	Retrospective case–control study linking vitamin D levels to infection risk.	Does not explore the impact on vaccine-induced immunity.
Kaya MO et al., 2021 [76]	Observed that vitamin D deficiency increases the risk of severe COVID-19 outcomes.	Meta-analysis emphasizing the protective role of adequate vitamin D levels.	Does not specifically examine post-vaccination immune responses.
Kaufman et al., 2020 [77]	Found that higher circulating 25-hydroxyvitamin D levels are associated with lower SARS-CoV-2 positivity rates.	Large-scale study suggesting vitamin D’s role in reducing infection risk.	Focuses on infection rates, not vaccine response.
Sulli et al., 2021 [78]	Reported that vitamin D levels influence lung outcomes in elderly COVID-19 patients.	Highlights the importance of vitamin D in respiratory health during COVID-19.	Does not address post-vaccination immunity.
Szarpak et al., 2021 [79]	Conducted a systematic review on the effect of vitamin D levels on COVID-19 incidence.	Provides a comprehensive overview of existing studies linking vitamin D to COVID-19.	Lacks focus on post-vaccination immune responses.
AlSafar et al., 2021 [80]	Found that vitamin D status correlates with COVID-19 disease severity and death among UAE residents.	Regional study emphasizing the role of vitamin D in COVID-19 outcomes.	Does not explore vaccine-induced immunity.
Chiodini et al., 2021 [81]	Observed that vitamin D deficiency increases the risk of severe COVID-19 outcomes.	Meta-analysis emphasizing the protective role of adequate vitamin D levels.	Does not specifically examine post-vaccination immune responses.

**Table 2:** Studies evaluating vitamin D, VDR, and COVID-19 vaccine-induced immune responses.

Researcher(s) and Year	Study Design	Vaccination Endpoint Evaluated	Major Findings	Strengths	Limitations
Shende and Rathored, 2024 **[82]**	Molecular observational study	VDR mRNA expression after COVID-19 vaccination	Demonstrated significant modulation of VDR mRNA expression following vaccination, suggesting involvement of VDR signaling in post-vaccination immune regulation.	One of the first molecular studies evaluating VDR expression after COVID-19 vaccination.	Small sample size; cross-sectional design; long-term immune responses not evaluated.
Cesur et al., 2023 **[5]**	Randomized controlled trial	SARS-CoV-2 IgG antibody response following vitamin D supplementation	Vitamin D_3_ supplementation significantly improved IgG antibody responses in vitamin D-deficient vaccinated women.	Randomized controlled design providing higher-quality clinical evidence.	Included only vitamin D-deficient women; findings may not be generalizable.
Abu Fanne et al., 2022 **[55]**	Prospective observational study	Humoral immunity and breakthrough infection	Baseline vitamin D levels positively correlated with post-vaccination antibody titers and lower breakthrough infection rates.	Simultaneously evaluated antibody response and clinical protection.	Observational design cannot establish causality.
di Filippo et al., 2023 **[60]**	Longitudinal cohort study	Long-term antibody persistence	Vitamin D deficiency predicted impaired long-term immune responses after COVID-19 vaccination.	Longitudinal follow-up assessing durability of vaccine immunity.	Single-center study with limited population diversity.
Meyers et al., 2023 **[61]**	Prospective cohort study	Antibody response following vaccination	No significant association was observed between baseline vitamin D status and vaccine-induced antibody response among nursing home residents and staff.	Included elderly high-risk population; standardized laboratory assays.	Population restricted to nursing homes; limited external validity.
Zelini et al., 2022 **[67]**	Observational cohort study	Humoral immune response (BNT162b2 vaccine)	Higher serum vitamin D concentrations were associated with stronger antibody responses following BNT162b2 vaccination.	Evaluated vaccine-specific antibody titers using standardized assays.	Observational design with potential confounding variables.
Chiu et al., 2021 **[54]**	Narrative review	Molecular mechanisms of vaccine immunogenicity	Summarized molecular mechanisms by which vitamin D and VDR signaling may regulate vaccine-induced adaptive immunity, cytokine balance, and antibody production.	Comprehensive mechanistic review integrating immunological evidence.	Narrative review; lacks original clinical data.
Jalali et al., 2020 **[83]**	Commentary	Serological testing following COVID-19	Discussed methodological challenges associated with interpreting post-vaccination serological responses.	Highlights limitations of antibody testing relevant to vaccine studies.	Commentary article without original experimental evidence.

## Data Availability

No new data were created or analyzed in this study. Data sharing is not applicable to this article.
